# Associations between the overall nutritional quality of prepackaged food categories consumed at breakfast or as snacks and the presence of nutrition-related labelling messages: a cross-sectional analysis of products sold in the province of Québec (Canada)

**DOI:** 10.1186/s40795-025-01186-z

**Published:** 2025-11-05

**Authors:** Annie Vézina, Alicia Corriveau, Mylène Turcotte, Clara-Jane Rhéaume, Véronique Provencher, Marie-Ève Labonté

**Affiliations:** 1 Centre Nutrition, Santé et Société (NUTRISS), Institute of Nutrition and Functional Foods (INAF), Université Laval, Pavillon des Services, 2440 Hochelaga Blvd, Québec, QC G1V 0A6 Canada; 2 School of Nutrition, Faculty of Agriculture and Food Science, Université Laval, Pavillon Paul-Comtois, 2425 Rue de l’Agriculture, Québec, QC G1V 0A6 Canada

**Keywords:** Nutrient profiling, Nutri-Score, Nutritional quality, Food supply, Food sales, Front-of-pack labelling, Nutrition claims, Food regulations, Healthfulness, Canada

## Abstract

**Background:**

In Canada, there are no requirements regarding the healthfulness of foods carrying nutrition claims. Also, as of 2026, most prepackaged foods with a high content in saturated fat, sugars and/or sodium will be required to display Health Canada’s front-of-pack symbol (HC-FOPS), regardless of their overall nutritional quality. This study aims to evaluate the associations between the overall nutritional quality of prepackaged foods and the presence of nutrition claims as well as of HC-FOPS on those foods.

**Methods:**

The score from -15 (more nutritious) to + 40 (less nutritious) or the accompanying grade from A to E generated by the Nutri-Score was used to assess the overall nutritional quality of four food categories from the Food Quality Observatory: Breakfast cereals (*n* = 310), Sliced breads (*n* = 261), Granola bars (*n* = 234), and Yogurts and dairy desserts (*n* = 279). Data were sales-weighted to better represent what consumers are buying.

**Results:**

In all categories, products with nutrition claims had a better overall nutritional quality (i.e. lower sales-weighted mean score) than products without claims (e.g. 8.00 ± 5.70 vs. 12.14 ± 4.41, respectively, for Breakfast cereals; -0.56 ± 3.01 vs. 1.31 ± 2.04 for Sliced breads; 10.16 ± 3.50 vs. 15.56 ± 5.00 for Granola bars; 0.43 ± 1.65 vs. 1.38 ± 2.70 for Yogurts and dairy desserts; all *p* ≤ 0.0004). Conversely, products which would be required to carry HC-FOPS generally had a lower overall nutritional quality than products which would not carry the symbol. However, additional analyses showed that some less nutritious foods (i.e. graded with letters D or E) carried nutrition claims or would not be required to display HC-FOPS, particularly in the categories of Granola bars (43.2% and 38.5%, respectively) and Breakfast cereals (23.8% and 11.8%).

**Conclusions:**

These findings show that while the presence of nutrition claims and the absence of HC-FOPS are generally indicative of a better overall nutritional quality, inconsistencies persist. This highlights the importance of strong educational campaigns to help consumers use nutrition-related labelling messages adequately. Results could also be used to support a more stringent regulatory framework for nutrition claims and HC-FOPS.

**Supplementary Information:**

The online version contains supplementary material available at 10.1186/s40795-025-01186-z.

## Background

In Canada, since 2003, most prepackaged foods are required to display a list of ingredients and a Nutrition Facts table to help consumers make informed choices at the grocery store [1]. Under current regulations, food manufacturers may also add nutrition claims on packages, which are optional statements highlighting favourable attributes related to nutrition and health [2]. These claims must meet predefined requirements to ensure they are accurate and not misleading. For example, to be labelled as a “source”, “good source” or “excellent source” of iron, a food product must contain at least 5%, 15% or 25% of the daily value (DV) for this mineral, respectively. Similarly, to be eligible to carry a claim about psyllium’s effect on bowel function, the amount of fibre from psyllium seed must be specified on the food product, that amount must be no less than 3.5 g/serving, and the claim’s statement must follow a prescribed wording. Aside from such claim-specific criteria, there is no requirement in Canada regarding the underlying overall nutritional quality (i.e. taking into account several nutrients simultaneously, including nutrients to be encouraged such as proteins or vitamins, and nutrients to be limited such as sugars or sodium) of foods carrying nutrition claims. In other words, a prepackaged food could, for example, carry a nutrition claim about iron or psyllium, despite having a relatively poor overall nutritional quality. This is concerning, as previous studies have shown that the presence of nutrition claims could mislead consumers by giving the impression that foods carrying claims are healthier than they really are [3–6].

Recently, a new component has been added to the Canadian labelling regulations. As of January 1^st^, 2026, most prepackaged foods high in saturated fat, sugars, or sodium will be required to display a Health Canada-endorsed front-of-pack (FOP) nutrition symbol featuring a magnifying glass, indicating that the products contain excessive amounts of one or more of these nutrients to limit [7]. This symbol is intended to help consumers identify foods high in saturated fat, sugars or sodium more easily, to limit their consumption, and ultimately reduce the risk of developing diet-related non-communicable diseases at the population level. The presence of one or more of these three nutrients in excess will trigger the symbol, regardless of the overall nutritional quality of a given food product. It will therefore be possible for foods of low overall nutritional quality to avoid carrying the symbol if their levels of saturated fat, sugars and sodium are all below the predetermined thresholds. Although this approach is similar to other FOP systems implemented over the last decade in some countries of the Americas (e.g. Argentina, Chile, Mexico, Peru, Uruguay, and Venezuela), it contrasts with other FOP symbols that consider not only nutrients to be limited, but also nutrients to be encouraged, such as the Nutri-Score (implemented in France, Belgium, Germany, Luxembourg, the Netherlands, Spain, and Switzerland) and the Health Star Rating system (in Australia and New Zealand) [8–10].

In parallel, breakfast and snacks are eating occasions that can be favourable to the consumption of nutritious foods, such as whole fruits, whole grains, and milk products [11–14]. These foods are important sources of nutrients that are generally insufficient in the diet of Canadians, such as fibre, potassium, magnesium, calcium, vitamin D, and vitamin A [15, 16]. The overall nutritional quality of prepackaged foods consumed at breakfast and as snacks can, however, be highly variable. Breakfast cereals, sliced breads, granola bars, and yogurts and dairy desserts are relevant examples, with nutritional values ranging from very high to very low within a same category [17]. For instance, sugar content can range from 0 to 31 g/serving and 5 to 28 g/serving, respectively, for breakfast cereals and granola bars sold in the province of Québec (Canada), while the sodium content of sliced breads can range from 0 to 620 mg/serving, and the saturated fat content of yogurts and dairy desserts from 0 to 16 g/serving [17]. Moreover, these food categories are among those with the highest prevalence of nutrition claims in Canada and elsewhere [18–23]. In this context, this study aims to evaluate the associations between the overall nutritional quality of prepackaged foods commonly consumed at breakfast or as a snack, and 1) the presence of nutrition claims, as well as 2) the theoretical presence of Health Canada’s FOP symbol (HC-FOPS).

## Methods

### Data sources and food categories included

Since 2016, the Food Quality Observatory (henceforth Observatory), from the Institute of Nutrition and Functional Foods (INAF) at Université Laval, monitors the nutritional quality of 15 prepackaged food categories sold in the province of Québec: Breakfast cereals, Pizzas, Ready-to-serve soups, Sliced breads, Sliced processed meats, Frozen meals, Granola bars, Pasta sauces, Yogurts and dairy desserts, Cookies, Sausages, Crackers, Salty snacks, Processed cheeses, as well as Plant-based beverages and flavoured milks [24]. These categories were selected by the Observatory’s Scientific and Knowledge Users Committees based on their potential health impact, the wide variability in their nutritional quality, their widespread presence in most Québec households, and their potential for improvement from a nutritional standpoint. For each of these food categories, all products found in supermarkets, warehouse club stores and specialty grocery stores are collected every 4 to 5 years [25, 26]. For each product, all sides of the packaging are photographed and information such as the Universal Product Code (UPC), the Nutrition Facts table, the list of ingredients, and logos are manually entered into an Excel file by two independent coders. Most of the products recorded by the Observatory are linked to sales volume data in kilograms from NielsenIQ using either the UPC or product name, which enables weighting nutritional composition data according to market shares [26]. The information from NielsenIQ is representative of the food sold in the province of Quebec and is mainly derived from optical scanning of products at checkouts.

The current study used the most recent data collections available for four food categories commonly consumed at breakfast or as a snack: Breakfast cereals, Sliced breads, Granola bars, and Yogurts and dairy desserts [12, 27–29]. These data collections all took place in the city of Québec and its surroundings between 2021 and 2023. Other characteristics of the data collections are presented in Supplementary Table 1.

### Assessment of the overall nutritional quality with the Nutri-Score

Nutrient profiling (NP) is considered as a transparent and reproducible method for assessing the overall nutritional quality of foods [30]. A few years ago, the Observatory set out to identify the most relevant NP models for characterizing and monitoring the nutritional quality of the food supply [31]. To guide this selection, rigorous criteria were established for the NP models, such as including both nutrients to encourage and nutrients to limit in their algorithm, in order to better reflect the food products’ overall nutritional quality; generating a numerical score (with or without subsequent classifications) to better discriminate between food products than NP models that solely generate classifications; and having been developed by academic, governmental or non-governmental institutions, rather than by commercial organizations (e.g. food manufacturers). In addition to meeting all of these criteria, the 2017 original Nutri-Score algorithm has recently been validated in the Canadian (Corriveau A., Harrison S., Turcotte M., Kayigire A.C., Provencher V., and Labonté M.È.: unpublished observations – manuscript under preparation) and Québec contexts, and a meta-analysis has shown that it has substantial criterion-related validity evidence in relation to health outcomes such as cardiovascular diseases, cancer and all-cause mortality [32, 33]. For these reasons, this NP model was selected to assess the overall nutritional quality of foods in the current study.

Developed in France, the Nutri-Score original algorithm (2017) takes into account a food’s content in protein, fibre, ingredients of vegetable origin (i.e. fruits, vegetables, nuts, legumes; FVNL), energy, saturated fat, sodium, and total sugars to generate a numerical score (henceforth referred to as “score” in the manuscript) ranging from −15 (highest overall nutritional quality) to + 40 (lowest overall nutritional quality) [9]. To help consumers make better choices when purchasing foods in some European countries, the scores generated in the category of “*general foods*” are then converted into one of five grades, represented by a colored letter from A (more nutritious) to E (less nutritious): dark green A for scores from −15 to −1; light green B from 0 to 2; yellow C from 3 to 10; orange D from 11 to 18; and dark orange E from 19 to 40. Letters are attributed differently for products considered as *“beverages”*: A for water only; B for scores from −15 to 1; C from 2 to 5; D from 6 to 9; and E from 10 to 40.

The nutritional data used to calculate the scores and corresponding grades were sourced from the Observatory’s databases. As Canadian regulations do not require quantitative ingredient declarations on prepackaged food labels, two independent coders estimated the percentage of FVNL in each product using a previously published method which considers the order in which FVNL ingredients appeared in the ingredient list [34]. Briefly, FVNL percentage was estimated to be ≤ 40% when a FVNL was not one of the first two ingredients; 41–60% when a FVNL was the second ingredient; 61–80% when a FVNL was the first ingredient, but non-FVNL ingredients appeared to contribute considerably to the weight of the product; or ≥ 81% when a FVNL was the first ingredient, and non-FVNL ingredients did not appear to contribute considerably to the weight of the product. When discrepancies were found between the two coders, a discussion was held to reach a consensus. When consensus could not be reached, a discussion was held with other team members to make a final decision. Scores and grades were also calculated by two independent coders using the Nutri-Score calculation tool, and all included products were considered as *“general foods”* according to the original algorithm [9, 35]. The coders’ scores and grades were identical for all products.

### Assessment of the presence of nutrition claims

All nutrition claims found on packaging were considered in the current study. In Canada, there are two main types of nutrition claims: nutrient content claims and health claims [36]. Health claims are further divided into disease risk reduction claims, function claims, general health claims, and implied health claims [2]. Supplementary Table 2 provides an overview of these nutrition claims, with examples. Using photos of all products in the four food categories, AV and AC independently coded the different types and subtypes of nutrition claims on the packaging, compared their results, discussed discrepancies, and were able to reach a consensus for all products. Claims related to composition and quality (e.g. 100% pure, no preservatives), method of production (e.g. organic, natural, minimally processed), origin (e.g. made in Canada) or allergens and gluten (e.g. gluten free, peanut-free symbol) were excluded from the present study as they are not recognized as nutrition claims per se under Canadian regulations [2]. Quantitative declarations which simply repeated data from the Nutrition Facts table (e.g. 70 cal per 30 g serving, 10% of the calcium DV per bar) were also excluded from the analyses.

### Identification of products required to carry HC-FOPS

Under the new Canadian FOP regulations, most prepackaged foods and non-alcoholic beverages that meet or exceed thresholds for one, two or three nutrients of concern (i.e. saturated fat, sugars and sodium) will be labelled with HC-FOPS [7]. These thresholds are based on the percent DV per reference amount for each nutrient. For foods with a reference amount ≤ 30 g or mL (e.g. puffed breakfast cereals, uncoated granola bars), the threshold is set at 10% of the DV for the three nutrients. For main dishes with a reference amount ≥ 200 g (e.g. pizza, frozen dinner), the threshold rises to 30% of the DV. For foods with a reference amount > 30 g or mL that are not meals (e.g. yogurts, sliced breads, coated granola bars, biscuit-type breakfast cereals), this threshold is set at 15% of the DV. Under certain conditions, some foods have been exempted from the requirement to display the FOP for saturated fat, sugars or sodium, including foods with recognized health protection benefits (e.g. unsweetened canned fruits, nuts with less than 30% of total fat from saturated fat), and foods which are top contributors of calcium (e.g. yogurts, cheese), given that calcium is a shortfall nutrient in the Canadian population.

Products that will be required to display HC-FOPS have been determined by CJR according to Canadian regulations available at the time of coding [37, 38]. Each food was first classified according to its reference amount in order to apply the appropriate corresponding DV threshold of 10% or 15% (the 30% threshold was not used as none of the included products were considered as main dishes) [39]. Then, if a food met or exceeded the DV threshold for saturated fat, sugars and/or sodium, it was determined that it would be required to carry HC-FOPS, unless it met the criteria for an exemption.

### Statistical analyses

All analyses are presented by food category and were conducted using R (version 4.3.2), with *p*-values < 0.05 considered statistically significant. Analyses were conducted using either the Nutri-Score numerical score or grade (letter from A to E). Using the *weights* argument in R, sales-weighted data were obtained by applying sales volumes in kilograms obtained from NielsenIQ to food supply data, to better represent what consumers are buying and eventually consuming. Wilcoxon rank sum tests were used to compare unweighted mean scores (results corresponding to food supply), and rank regressions were performed to compare sales-weighted mean scores (results corresponding to food sales). Unweighted (food supply) and weighted (food sales) Spearman’s correlation coefficients were calculated to assess the alignment between scores and the presence of nutrition-related labelling messages, which include nutrition claims and HC-FOPS.

## Results

### Nutrition claims

As shown in Table 1, based on food sales data, between 55.6% and 76.1% of products in all four food categories carried at least one nutrition claim, regardless of the type or subtype of claim. For Breakfast cereals, Sliced breads and Granola bars, nutrient content claims were the most prevalent (48.7–66.5%), followed by general health claims (19.2–41.0%), implied health claims (3.0–12.6%), and very few disease risk reduction and function claims (0–6.1%). For Yogurts and dairy desserts, nutrient content claims were also the most prevalent (54.8%), followed this time by implied health claims (37.6%), function claims (28.3%), general health claims (16.8%), and disease risk reduction claims, which appeared on none of the products (0%). Similar results were observed when looking at food supply data.

**Table 1:** Number and percentage of products with nutrition claims^a^ or which will have to display HC-FOPS^b^

**Food supply**^c^** or food sales**^d^	**Food category**	**At least one nutrition claim**		**Nutrient content claim**		**Disease risk reduction claim**		**Function claim**		**General health claim**		**Implied health claim**		**Presence of HC-FOPS**		**HC-FOPS for 1 nutrient**			**HC-FOPS for 2 nutrients**			**At least one nutrition claim AND presence of HC-FOPS**	
		***n***	**% of total**	***n***	**% of total**	***n***	**% of total**	***n***	**% of total**	***n***	**% of total**	***n***	**% of total**	***n***	**% of total**	***n***	**% of total**	**% of products with HC-FOPS**	***n***	**% of total**	**% of products with HC-FOPS**	***n***	**% of total**
Supply	Breakfast cereals *n* = 392	311	79.3	272	69.4	18	4.6	6	1.5	160	40.8	43	11.0	121	30.9	99	25.3	81.8	22	5.6	18.2	94	24.0
	Sliced breads *n* = 340	217	63.8	186	54.7	9	2.6	20	5.9	115	33.8	24	7.1	125	36.8	121	35.6	96.8	4	1.2	3.2	59	17.4
	Granola bars *n* = 369	220	59.6	200	54.2	0	0.0	3	0.8	71	19.2	23	6.2	182	49.3	144	39.0	79.1	38	10.3	20.9	108	29.3
	Yogurts and dairy desserts *n* = 387	255	65.9	210	54.3	0	0.0	94	24.3	47	12.1	120	31.0	73	18.9	61	15.8	83.6	12	3.1	16.4	40	10.3
Sales	Breakfast cereals *n* = 310	236	76.1	206	66.5	16	5.2	4	1.3	127	41.0	39	12.6	83	26.8	73	23.5	88.0	10	3.2	12.0	57	18.4
	Sliced breads *n* = 261	180	69.0	151	57.9	9	3.4	16	6.1	102	39.1	20	7.7	91	34.9	89	34.1	97.8	2	0.8	2.2	53	20.3
	Granola bars *n* = 234	130	55.6	114	48.7	0	0.0	0	0.0	45	19.2	7	3.0	100	42.7	79	33.8	79.0	21	9.0	21.0	53	22.6
	Yogurts and dairy desserts *n* = 279	191	68.5	153	54.8	0	0.0	79	28.3	47	16.8	105	37.6	49	17.6	45	16.1	91.8	4	1.4	8.2	29	10.4

^a^An overview of the different types of nutrition claims is available in Supplementary Table 2

^b^Health Canada’s front-of-pack symbol

^c^When considering products found on the store shelves by the Observatory

^d^When considering products found on the shelves and matched with a product from the NielsenIQ database (i.e. products with sales volume data)

Table 2 shows that based on food sales data, products with at least one nutrition claim had a better overall nutritional quality (i.e. lower sales-weighted mean score) than products without claims within each of the four food categories analyzed (i.e. 8.00 ± 5.70 vs. 12.14 ± 4.41, respectively, for Breakfast cereals; −0.56 ± 3.01 vs. 1.31 ± 2.04 for Sliced breads; 10.16 ± 3.50 vs. 15.56 ± 5.00 for Granola bars; 0.43 ± 1.65 vs. 1.38 ± 2.70 for Yogurts and dairy desserts; all *p* ≤ 0.0004). Similar results were observed when considering food supply data (all *p* ≤ 0.01). Further analyses using Spearman’s correlations (Table 3) with both food supply and food sales data showed inverse associations between the score and the presence of at least one nutrition claim in all four food categories (*r* between −0.13 and −0.49, all *p* < 0.05), again suggesting a better overall nutritional quality for products carrying claims. Similar results were observed when specifically looking at correlations between the score and the presence of a nutrient content claim within each food category (*r* between −0.23 and −0.44, all *p* < 0.05). Contrasting results were however observed when looking at the different sub-types of health claims. Correlations between the score and the presence of disease risk reduction claims, function claims, general health claims or implied health claims were often weak or not statistically significant. Two positive associations have also been observed between the score and the presence of a specific sub-type of health claim, namely general health claims in Breakfast cereals (*r* + 0.17, *p* = 0.0032, when using food sales data), as well as function claims in Granola bars (*r* + 0.13, *p* = 0.014, when using food supply data). These positive associations suggest that the presence of some specific types of health claims in Breakfast cereals and Granola bars reflects a lower overall nutritional quality.

**Table 2:** Comparison of the mean score^a^ of products with and without nutrition claims^b^

**Food supply**^c^** or food sales**^d^	**Food category**	**Products with nutrition claims**			**Products without nutrition claims**			**Difference**^e^	***p*-value**^f^
		***n***	**Mean score**	**Standard deviation**	***n***	**Mean score**	**Standard deviation**		
Supply	Breakfast cereals	311	5.86	5.86	81	9.22	6.47	−3.36	< 0.0001
	Sliced breads	217	−1.44	2.99	123	1.35	3.63	−2.79	< 0.0001
	Granola bars	220	9.22	4.85	149	14.58	5.10	−5.36	< 0.0001
	Yogurts and dairy desserts	255	0.63	1.86	132	2.22	4.65	−1.59	0.01
Sales	Breakfast cereals	236	8.00	5.70	74	12.14	4.41	−4.14	< 0.0001
	Sliced breads	180	−0.56	3.01	81	1.31	2.04	−1.87	< 0.0001
	Granola bars	130	10.16	3.50	104	15.56	5.00	−5.40	< 0.0001
	Yogurts and dairy desserts	191	0.43	1.65	88	1.38	2.70	−0.95	0.0004

^a^Numerical score generated by the Nutri-Score algorithm version 2017, where a lower score reflects a better overall nutritional quality

^b^An overview of the different types of nutrition claims is available in Supplementary Table 2

^c^When considering products found on the store shelves by the Observatory

^d^When considering products found on the shelves and matched with a product from the NielsenIQ database (i.e. products with sales volume data)

^e^Difference calculated by subtracting the mean score of products without nutrition claims from the mean score of products with nutrition claims. A negative result means that the mean score of products with nutrition claims reflects a better overall nutritional quality than that of products without claims. In contrast, a positive result means that the mean score of products with nutrition claims reflects a lower overall nutritional quality than that of products without claims

^f^According to the Wilcoxon rank sum test (food supply) or rank regressions (food sales); *p*-values < 0.05 considered statistically significant

**Table 3:** Spearman’s correlation coefficients between the score^a^ and the presence of nutrition claims or HC-FOPS^c^

**Food supply**^d^** or food sales**^e^	**Food category**	**At least one nutrition claim**	**Nutrient content claim**	**Disease risk reduction claim**	**Function claim**	**General health claim**	**Implied health claim**	**HC-FOPS**	**At least one nutrition claim—If claims were removed from products with HC-FOPS**
Supply	Breakfast cereals	−0.24^*^	−0.24^*^	−0.03	−0.01	−0.12^*^	+ 0.03	+ 0.60^*^	−0.57^*^
	Sliced breads	−0.42^*^	−0.44^*^	−0.08	−0.16^*^	−0.37^*^	−0.17^*^	+ 0.47^*^	−0.52^*^
	Granola bars	−0.45^*^	−0.43^*^	N/A	+ 0.13^*^	−0.25^*^	−0.21^*^	+ 0.42^*^	−0.49^*^
	Yogurts and dairy desserts	−0.13^*^	−0.23^*^	N/A	−0.09	−0.002	−0.004	+ 0.43^*^	−0.21^*^
Sales	Breakfast cereals	−0.27^*^	−0.30^*^	+ 0.01	−0.08	+ 0.17^*^	−0.006	+ 0.65^*^	−0.64^*^
	Sliced breads	−0.25^*^	−0.37^*^	−0.19^*^	−0.03	−0.42^*^	−0.20^*^	+ 0.27^*^	−0.42^*^
	Granola bars	−0.49^*^	−0.40^*^	N/A	N/A	−0.15^*^	−0.25^*^	+ 0.42^*^	−0.48^*^
	Yogurts and dairy desserts	−0.19^*^	−0.31^*^	N/A	−0.04	+ 0.08	+ 0.001	+ 0.07	−0.13^*^

N/A: not applicable (i.e. no product with the corresponding nutrition claim)

^a^Numerical score generated by the Nutri-Score algorithm version 2017, where a lower score reflects a better overall nutritional quality

^b^An overview of the different types of nutrition claims is available in Supplementary Table 2

^c^Health Canada’s front-of-pack symbol

^d^When considering products found on the store shelves by the Observatory

^e^When considering products found on the shelves and matched with a product from the NielsenIQ database (i.e. products with sales volume data)

^*^Significant correlations (*p*-values < 0.05) according to unweighted (food supply) and weighted (food sales) Spearman’s correlation

**Fig. 1:**
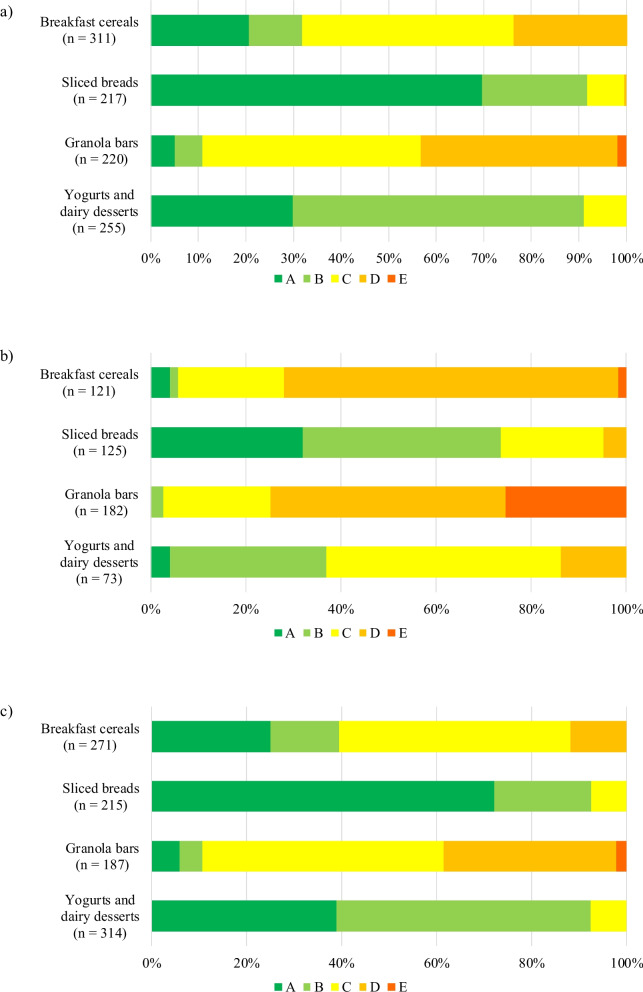
Nutri-Score (version 2017) grade distribution for products offered on the market (food supply). Panel **a**) shows grade distribution for products carrying at least one nutrition claim (an overview of the different types of nutrition claims is available in Supplementary Table 2). Panel **b**) shows grade distribution for products which would have to carry Health Canada’s front-of-pack symbol (HC-FOPS). Panel **c)** shows grade distribution for products which would not carry HC-FOPS. The specific number and percentage of products with and without nutrition claims and HC-FOPS, by grade, are available in Supplementary Table 3

Analyses were also conducted using the Nutri-Score grades. As shown in Fig. 1a, in all food categories, most products available in the food supply that carry at least one nutrition claim would be assigned a grade from A to C. However, 23.8% of Breakfast cereals (*n* = 74), 0.5% of Sliced breads (*n* = 1), and 41.4% of Granola bars (*n* = 91) with at least one nutrition claim would be graded D. Furthermore, 1.8% of Granola bars (*n* = 4) carrying nutrition claims would be graded E. Supplementary Table 3 provides more details about the number and percentage of products with nutrition claims, by grade.

### Health Canada’s FOP symbol

Some products in the four food categories would have to carry HC-FOPS, ranging from 17.6% of products in Yogurts and dairy desserts to 42.7% of products in Granola bars, according to sales data (Table 1). Among these products, most would have the symbol for only one nutrient (79.0–97.8%), a smaller proportion for two nutrients (2.2–21.0%), and none for all three nutrients simultaneously (data not shown).

As shown in Table 4, products which would be required to carry HC-FOPS had a lower overall nutritional quality (i.e. higher mean score) than products which would not be required to carry the symbol within each food category, except for Yogurts and dairy desserts when using sales-weighted data (*p* = 0.18 vs. *p* < 0.0001 in all other cases). Results from the correlation analysis between the score and the presence of HC-FOPS are similar (Table 3).

**Table 4:** Comparison of the mean score^a^ of products with and without HC-FOPS^b^

**Food supply**^c^** or food sales**^d^	**Food category**	**Products with HC-FOPS**			**Products without HC-FOPS**			**Difference**^e^	***p-*value**^f^
		***n***	**Mean score**	**Standard deviation**	***n***	**Mean score**	**Standard deviation**		
Supply	Breakfast cereals	121	11.74	4.39	271	4.23	5.33	7.51	< 0.0001
	Sliced breads	125	1.71	3.83	215	−1.67	2.58	3.38	< 0.0001
	Granola bars	182	13.77	5.39	187	9.07	4.78	4.70	< 0.0001
	Yogurts and dairy desserts	73	4.64	4.69	314	0.37	2.02	4.27	< 0.0001
Sales	Breakfast cereals	83	13.85	2.43	227	6.70	5.37	7.15	< 0.0001
	Sliced breads	91	1.57	2.58	170	−0.61	2.73	2.18	< 0.0001
	Granola bars	100	15.48	5.28	134	11.06	3.98	4.42	< 0.0001
	Yogurts and dairy desserts	49	1.35	2.36	230	0.57	1.98	0.78	0.18

^a^Numerical score generated by the Nutri-Score algorithm version 2017, where a lower score reflects a better overall nutritional quality

^b^Health Canada’s front-of-pack symbol

^c^When considering products found on the store shelves by the Observatory

^d^When considering products found on the shelves and matched with a product from the NielsenIQ database (i.e. products with sales volume data)

^e^Difference calculated by subtracting the mean score of products without HC-FOPS from the mean score of products with HC-FOPS. A negative result means that the mean score of products with HC-FOPS reflects a better overall nutritional quality than that of products without HC-FOPS. In contrast, a positive result means that the mean score of products with HC-FOPS reflects a lower overall nutritional quality than that of products without HC-FOPS

^f^According to the Wilcoxon rank sum test (food offer) or rank regressions (food sales); *p*-values < 0.05 considered statistically significant

As shown in Fig. 1b and Supplementary Table 3, most of the Breakfast cereals (71.9%, *n* = 87) and Granola bars (74.7%, *n* = 136) which would be required to carry HC-FOPS would be assigned grades of D or E. In the case of Sliced breads and Yogurts and dairy desserts, a minority of products (4.8%, *n* = 6 and 13.7%, *n* = 10, respectively) which would carry the symbol would simultaneously be given a grade D, and none would be given a grade E. Supplementary Table 3 also shows that 5.8% of Breakfast cereals (*n* = 7), 73.6% of Sliced breads (*n* = 92), 2.7% of Granola bars (*n* = 5), as well as 37.0% of Yogurts and dairy desserts (*n* = 27) offered on the market which would carry HC-FOPS would be assigned grades of A or B.

On the other hand, Fig. 1c presents the grade distribution for products which would not be required to carry HC-FOPS. All of these products in the Sliced breads as well as Yogurts and dairy desserts categories would have a grade ranging from A to C. For the other two food categories, although most of their products would be graded from A to C, 11.8% of Breakfast cereals (*n* = 32) would be graded D, and 38.5% of Granola bars (*n* = 72) would be graded D or E (Supplementary Table 3).

As shown in Table 1, according to food sales data, between 10.4% and 22.6% of products in all four food categories would carry both nutrition claims and HC-FOPS. Interestingly, a sub-analysis in which nutrition claims would hypothetically be removed from products carrying HC-FOPS (Table 3) showed that the strength of the inverse associations between the score and the presence of at least one nutrition claim increased for Breakfast cereals (e.g. *r* from −0.27 to −0.64 for food sales, all *p* < 0.05) and Sliced breads (e.g. *r* from −0.25 to −0.42 for food sales, all *p* < 0.05). In other words, in these food categories, the presence of nutrition claims was more strongly associated with a better overall nutritional quality (i.e. lower score) when nutrition claims hypothetically remained only on foods that would not display HC-FOPS. Similar results were observed when using food supply data.

## Discussion

This study examined the associations between the overall nutritional quality estimated using the 2017 algorithm of the Nutri-Score NP model, and the presence of nutrition-related labelling messages, namely nutrition claims and HC-FOPS, in Breakfast cereals, Sliced breads, Granola bars, as well as Yogurts and dairy desserts sold in grocery stores in the province of Québec, Canada.

### Nutrition claims

The presence of nutrition claims was associated with a better overall nutritional quality in the four prepackaged food categories analyzed. This favourable association appeared to be primarily driven by the presence of nutrient content claims on food products. In contrast, correlations between the overall nutritional quality and the presence of different sub-types of health claims on food products were either weaker, non-significant, or even positive in some cases. Analyses based on the Nutri-Score grade further showed that some less nutritious products offered on the market (i.e. graded D or E), especially within Breakfast cereals and Granola bars, carried nutrition claims highlighting positive attributes.

Similar results were found in previous Canadian, Brazilian and European studies that also used a NP model which included nutrients and components both to encourage and to limit [3, 18, 19, 40, 41]. These studies showed that prepackaged foods with nutrition claims had, on average, a better overall nutritional quality than foods without claims, although several products carrying claims did not have a favourable nutritional profile. Such observations however contrast with a study conducted in Spain, which reported that the overall nutritional quality of foods with protein claims was lower than that of foods without such claims [42]. The contrasting results between the Spanish study and the current one might be explained, at least in part, by the fact that the Spanish study only included claims referring to a high protein content, that the NP model they used only considered nutrients to limit, and that they analyzed a higher number of food categories (i.e. bars; biscuits; breads; breakfast cereals; cereal cakes/crackers; fruit drinks; milk substitutes; plant-based meat analogues; toasted bread; yogurts/dairy dessert substitutes; and yogurts/fermented milk) than in the current study [42]. Interestingly, researchers from INFORMAS Canada recently used HC-FOPS as a way to evaluate the nutritional quality of products carrying nutrition claims in INFORMAS-defined food categories (i.e. breakfast cereals; cookies and granola bars; dairy and plant-based milks; salty snacks and crackers; and dairy and plant-based yogurts and kefirs), also using data originating from the Observatory’s databases [43]. Although this NP model only considers nutrients to limit, the authors found that products with nutrition claims were less likely to be required to display HC-FOPS, an observation which is in line with results observed in similar food categories in the current study, generated using the Nutri-Score algorithm.

A growing body of evidence suggests that foods carrying nutrition claims are often perceived by consumers as being more nutritious than they actually are [3–6, 42]. To address this, Australia and New Zealand introduced the Nutrient Profiling Scoring Criterion (NPSC) to determine which foods and beverages could carry nutrition claims [21, 44]. Similar to the Nutri-Score, the NPSC is a NP model that assesses the overall nutritional quality of products by considering nutrients that should be limited (i.e. energy, saturated fat, total sugars, and sodium), along with nutrients and components that should be encouraged (i.e. protein, fibre, and FVNL). Under labelling regulations in Australia and New Zealand, nutrition claims can only be applied on foods and beverages that reach a certain NPSC score threshold. If this threshold is not reached, nutrition claims cannot be made on the product. It would currently be challenging to apply a similar regulation in Canada, particularly given that ingredient lists do not allow to determine precisely the proportion of ingredients coming from FVNL, one of the seven components considered in the NPSC algorithm. Canada could however adopt a regulation similar to Argentina or Mexico, where products that have to display a nutrient-specific FOP symbol similar to HC-FOPS cannot carry nutrition claims [45]. This approach has actually been recently proposed in two Canadian food policy reports advocating for healthier food environments in the country, as a way to avoid sending conflicting information to consumers [43, 46]. Results of the present study even showed that it was possible, in some cases, to improve the strength of the correlation between the overall nutritional quality and the presence of nutrition claims on food products by hypothetically removing claims from the products that would have to carry HC-FOPS.

### Health Canada’s FOP symbol

This study also showed that the presence of HC-FOPS was associated with a lower overall nutritional quality as measured by the Nutri-Score algorithm in all four prepackaged food categories, except for Yogurts and dairy desserts when specifically using sales-weighted data. The lack of association between the score and HC-FOPS when considering food sales for Yogurts and dairy desserts could be partly attributable to the fact that products available on the market which will be required to carry this nutrition symbol and for which sales data were available had a better overall nutritional quality (i.e. lower mean score) than their equivalent without sales data (i.e. *n* = 49, 3.45 ± 3.28 vs. *n* = 24, 7.08 ± 6.09, respectively; *p* = 0.017). In addition, Yogurts and dairy desserts that would not be required to carry the nutrition symbol and for which sales data were available had a lower overall nutritional quality (i.e. higher mean score) compared to their equivalent without sales data (i.e. *n* = 230, 0.60± 2.01 vs. *n* = 84, −0.29 ± 1.90, respectively; *p* = 0.0008). Given that sales-weighted analyses only consider products for which sales data are available, products collected by the Observatory that were not found in the NielsenIQ databases have been removed. This might have contributed to reducing the gap between the scores of products with and without HC-FOPS when food sales were considered.

Additional analyses have shown that certain products in the four food categories, considered to be the most nutritious according to the Nutri-Score grades (letters A or B), would have to display HC-FOPS. This could be explained by high levels of saturated fat, sugars and/or sodium in these products being offset by high levels of beneficial nutrients and components (i.e. protein, fibre, and FVNL), resulting in a more favourable overall nutritional quality as measured with the Nutri-Score. In a recent position paper from the Global Health Advocacy Incubator, such observations were actually raised as a concern of NP scoring systems that integrate nutrients and components to be encouraged in addition to those to be limited (e.g. Nutri-Score, Health Star Rating), because the numerical scores generated by such NP models do not allow identifying easily the levels of individual nutrients to be limited [45]. On the other hand, the current observations also suggest that HC-FOPS may be of interest for consumers who need to reduce their intake of either saturated fat, sugars or sodium because of a specific health condition (e.g. reducing sodium in people with hypertension), despite the relatively good overall nutritional quality of certain products.

To our knowledge, this was the first study in the country to specifically assess the association between an indicator of overall nutritional quality which considers both nutrients to limit and to encourage, and HC-FOPS, which focuses only on nutrients to be limited, within a sample of prepackaged foods. Nevertheless, a previous study has examined the diet quality of Canadian adults using different dietary index scores underpinning certain NP models [47]. The authors found that a dietary index based on HC-FOPS rated the diet quality of the population as healthier than dietary indexes deriving from other NP models, including the Nutri-Score. Furthermore, the dietary index derived from HC-FOPS showed a moderate association with the Nutri-Score dietary index (*r* = + 0.44) [47]. Combined with the results of the current study, which demonstrated that some products considered among the least nutritious (i.e. grade D or E) would not be required to carry HC-FOPS, this suggests that finding ways to strengthen the association between the overall nutritional quality of foods and the presence of HC-FOPS would be relevant. This could be achieved, for example, by setting stricter nutrient thresholds triggering HC-FOPS in certain food categories such as Breakfast cereals and Granola bars. This might contribute to reducing the number of products with a less favourable overall nutritional quality that would be free from carrying HC-FOPS.

### Strengths and limitations

This study has several key strengths. These include using a NP model previously validated in the country, weighting data according to sales volumes to better represent what consumers are buying, the fact that most variables analyzed were entered by two independent coders for greater data accuracy, and the large sample of products available in the Observatory’s databases. This study also has certain limitations. First, the percentage of FVNL ingredients used to calculate the overall nutritional quality score had to be estimated using a surrogate method developed by Canadian researchers [34]. Although rigorous and reproducible, this method is not as accurate as quantitative ingredient declarations. Second, the original 2017 version of the Nutri-Score algorithm was used in the current study although it was updated in late 2023 [35, 48]. Recent evidence indicates that the updated version better aligns than the previous version with France’s dietary guidelines [49]. Similar results could likely be observed in Canada, as dietary guidelines are relatively comparable between the two countries. However, the updated algorithm was not available at the time the research team conducted the vality testing studies in the Canadian (Corriveau A., Harrison S., Turcotte M., Kayigire A.C., Provencher V., and Labonté M.È.: unpublished observations – manuscript under preparation) and Québec contexts, which explains why the original version was used in the present manuscript [32]. Third, the presence of HC-FOPS was not mandatory yet at the time of the data collections. This means that the proportion of products predicted to carry this symbol in the current study may therefore be an overestimate of the actual proportion of products that will be seen on store shelves starting in 2026. Indeed, it is possible that some products are currently being reformulated to avoid the FOP once it becomes mandatory. Furthermore, the nutritional data used in this study are from 2021 for Breakfast cereals and Sliced breads, and from 2023 for Granola bars and Yogurts and dairy desserts. Thus, findings do not cover product changes that might have occurred in the food supply in very recent years. Finally, the trends observed in this study, which focused on four food categories, cannot be generalized to all prepackaged foods.

## Conclusions

This study showed that prepackaged food products often consumed at breakfast or as snacks and carrying nutrition claims tended to have a better overall nutritional quality than products which did not carry claims. With respect to HC-FOPS, products which would be required to carry the symbol generally had a lower overall nutritional quality than products which would avoid this symbol as of January 2026. While such results are reassuring from a public health perspective, it was also found that some foods of low overall nutritional quality do not follow the above trends, meaning that they still carry nutrition claims or that they would potentially avoid the display of HC-FOPS. This could send conflicting messages to consumers, therefore highlighting the importance of strong educational campaigns to help consumers use these nutrition-related labelling messages adequately. The current findings could also be used to support a more stringent regulatory framework for nutrition claims and HC-FOPS. This could be achieved by not allowing foods that will be required to carry HC-FOPS to make nutrition claims, or by setting stricter thresholds for saturated fat, sugars and sodium in some food categories when determining which foods will have to display this symbol. More research is warranted to determine whether the patterns observed here in Breakfast cereals, Sliced breads, Granola bars, and Yogurts and dairy desserts, are also reflected in other prepackaged food categories, and whether they persist over time, especially once HC-FOPS becomes mandatory.

## Supplementary Information

Supplementary Material 1.

## Data Availability

The datasets analysed during the current study are available from the corresponding author on reasonable request, but restrictions apply to sales volume data due to agreement signed with NielsenIQ.

## Abbreviations

DV: Daily value
FOP: Front-of-pack
FVNL: Fruits, vegetables, nuts, legumes
HC-FOP: Health Canada’s front-of-pack symbol
INAF: Institute of Nutrition and Functional Foods
NP: Nutrient profiling
NPSC: Nutrient profiling Scoring Criterion
UPC: Universal Product Code
