# Supplementary material for: Associations between the overall nutritional quality of prepackaged food categories consumed at breakfast or as snacks and the presence of nutrition-related labelling messages: a cross-sectional analysis of products sold in the province of Québec (Canada)
Source: BMC Nutr. 2025 Nov 5;11:204. doi: 10.1186/s40795-025-01186-z (PMC12590596; doi:10.1186/s40795-025-01186-z)
Supplement: Supplementary file 1 — Supplementary Material 1. [file 40795_2025_1186_MOESM1_ESM.docx]

**Additional file 1**

**Supplementary Table 1** Data collection characteristics for Breakfast cereals, Sliced breads, Granola bars, and Yogurts and dairy desserts

| **Characteristics** | **Breakfast cereals** | **Sliced breads** | **Granola bars** | **Yogurts and dairy desserts** |
| --- | --- | --- | --- | --- |
| **Year** | 2021 | 2021 | 2023 | 2023 |
| ***n* food supply**^a^ | 392 | 340 | 369 | 387 |
| ***n* food sales**^b^ | 310 | 261 | 234 | 279 |
| **% products with sales data**^c^ | 79% | 77% | 63% | 72% |
| **% market coverage**^d^ | 93% | 80% | 74% | 89% |
| **Included products** | Ready-to-eat breakfast cereals in an individual package | Products in a package with a Nutrition Facts table and available in grocery stores | Products sold in a box | Yogurts, fresh cheeses, puddings, cream desserts and dessert substitutes sold in multipack or individual containers, generally consumed on a single occasion |
| **Excluded products** | Multipacks including several varieties, hot cereals (e.g. oatmeal), dry infant cereals | Bagels, pitas, baguettes, etc. | Products sold in individual format, meal replacement bars | Family-size containers (e.g. 650 g), frozen desserts or puddings containing no dairy or plant-based alternatives |

^a^ Number of products found on the store shelves by the Observatory.

^b^ Number of products with sales volume data (i.e. products found on the shelves and matched to a product in the NielsenIQ database).

^c^ Percentage of products with sales volume data, out of the total number of products found on the store shelves by the Observatory.

^d^ Products with sales volume data identified by the Observatory, in proportion to all products listed in the NielsenIQ database for a given food category.

**Supplementary Table 2** Overview of nutrition claims in Canada^a^

| **Type** | **Subtype** | **Description** | **Examples** |
| --- | --- | --- | --- |
| Nutrient content claims | N/A | Describe the amount of a specific nutrient in a food | - “High source of fibres” - “Low in fat” - “Reduced in sugars” - “Excellent source of iron” - “Source of 6 essential nutrients” |
| Health claims | Disease risk reduction claims | Describe the relationship between the consumption of a nutrient or ingredient and the risk of developing a disease or condition | - “A healthy diet rich in a variety of vegetables and fruit may help reduce the risk of heart disease” - “A healthy diet with adequate calcium and vitamin D, and regular physical activity, help to achieve strong bones and may reduce the risk of osteoporosis” - “Psyllium fibre helps lower cholesterol” |
|  | Function claims | Describe the benefits of consuming a nutrient or ingredient on normal body functions | - “One serving of this product contains 3.5 grams of fibre from psyllium seed, which promotes regularity” - “Vitamin A contributes to the normal function of the immune system” - “With probiotics that contribute to healthy gut flora” |
|  | General health claims | Broad claims promoting health through a healthy diet or providing dietary guidance | - “Made with nutritious ingredients” - “Good for you” - “One serving of this product contains 12 grams of plant-based protein. Canada’s Food Guide recommends eating plant-based protein more often.” - “Healthy snack” |
|  | Implied health claims | Logos, symbols or words that contribute to the overall impression given by a food | - A heart-shaped icon implying that a product has a positive impact on cardiovascular health - Use of words such as “probiotics” or “antioxidants” |

^a^ According to the Industry Labelling Tool from the Canadian Food Inspection Agency (1).

N/A: not applicable.

**Supplementary Table 3** Number and percentage of products with and without nutrition-related labelling messages, by grade (food supply)^a^

| **Food category** | **Labelling message** | **A** | | **B** | | **C** | | **D** | | **E** | |
| --- | --- | --- | --- | --- | --- | --- | --- | --- | --- | --- | --- |
|  |  | ***n*** | **%** | ***n*** | **%** | ***n*** | **%** | ***n*** | **%** | ***n*** | **%** |
| Breakfast cereals | Overall  *n* = 392 | 73 | 18.6 | 41 | 10.5 | 159 | 40.6 | 117 | 29.8 | 2 | 0.5 |
|  | With nutrition claims  *n* = 311 | 64 | 20.6 | 35 | 11.3 | 138 | 44.4 | 74 | 23.8 | 0 | 0.0 |
|  | Without nutrition claims  *n* = 81 | 9 | 11.1 | 6 | 7.4 | 21 | 25.9 | 43 | 53.1 | 2 | 2.5 |
|  | With HC-FOPS  *n* = 121 | 5 | 4.1 | 2 | 1.7 | 27 | 22.3 | 85 | 70.2 | 2 | 1.7 |
|  | Without HC-FOPS  *n* = 271 | 68 | 25.1 | 39 | 14.4 | 132 | 48.7 | 32 | 11.8 | 0 | 0.0 |
| Sliced breads | Overall  *n* = 340 | 195 | 57.4 | 96 | 28.2 | 43 | 12.6 | 6 | 1.8 | 0 | 0.0 |
|  | With nutrition claims  *n* = 217 | 151 | 69.6 | 48 | 22.1 | 17 | 7.8 | 1 | 0.5 | 0 | 0.0 |
|  | Without nutrition claims  *n* = 123 | 44 | 35.8 | 48 | 39.0 | 26 | 21.1 | 5 | 4.1 | 0 | 0.0 |
|  | With HC-FOPS  *n* = 125 | 40 | 32.0 | 52 | 41.6 | 27 | 21.6 | 6 | 4.8 | 0 | 0.0 |
|  | Without HC-FOPS  *n* = 215 | 155 | 72.1 | 44 | 20.5 | 16 | 7.4 | 0 | 0.0 | 0 | 0.0 |
| Granola bars | Overall  *n* = 369 | 11 | 3.0 | 14 | 3.8 | 136 | 36.9 | 158 | 42.8 | 50 | 13.6 |
|  | With nutrition claims  *n* = 220 | 11 | 5.0 | 13 | 5.9 | 101 | 45.9 | 91 | 41.4 | 4 | 1.8 |
|  | Without nutrition claims  *n* = 149 | 0 | 0.0 | 1 | 0.7 | 35 | 23.5 | 67 | 45.0 | 46 | 30.9 |
|  | With HC-FOPS  *n* = 182 | 0 | 0.0 | 5 | 2.7 | 41 | 22.5 | 90 | 49.5 | 46 | 25.3 |
|  | Without HC-FOPS  *n* = 187 | 11 | 5.9 | 9 | 4.8 | 95 | 50.8 | 68 | 36.4 | 4 | 2.1 |
| Yogurts and dairy desserts | Overall  *n* = 387 | 125 | 32.3 | 192 | 49.6 | 60 | 15.5 | 10 | 2.6 | 0 | 0.0 |
|  | With nutrition claims  *n* = 255 | 76 | 29.8 | 156 | 61.2 | 23 | 9.0 | 0 | 0.0 | 0 | 0.0 |
|  | Without nutrition claims  *n* = 132 | 49 | 37.1 | 36 | 27.3 | 37 | 28.0 | 10 | 7.6 | 0 | 0.0 |
|  | With HC-FOPS  *n* = 73 | 3 | 4.1 | 24 | 32.9 | 36 | 49.3 | 10 | 13.7 | 0 | 0.0 |
|  | Without HC-FOPS  *n* = 314 | 122 | 38.9 | 168 | 53.5 | 24 | 7.6 | 0 | 0.0 | 0 | 0.0 |

^a^ According to the food supply data which consider all products found on the store shelves by the Observatory.

**Reference:**

1. Canadian Food Inspection Agency. Food labelling for industry. Available online: <https://inspection.canada.ca/en/food-labels/labelling/industry> (accessed March 17, 2025). Date modified: 2025-01-15.
